# Transcranial Direct Current Stimulation (tDCS) for Depression during Pregnancy: Scientific Evidence and What Is Being Said in the Media—A Systematic Review

**DOI:** 10.3390/brainsci8080155

**Published:** 2018-08-14

**Authors:** Anna Katharina Kurzeck, Beatrice Kirsch, Elif Weidinger, Frank Padberg, Ulrich Palm

**Affiliations:** Department of Psychiatry and Psychotherapy, Klinikum der Universität München, Ludwig-Maximilian University, 80336 Munich, Germany; annakatharina.kurzeck@t-online.de (A.K.K.); beatrice.kirsch@med.uni-muenchen.de (B.K.); elif.weidinger@med.uni-muenchen.de (E.W.); frank.padberg@med.uni-muenchen.de (F.P.)

**Keywords:** tDCS, transcranial direct current stimulation, non-invasive brain stimulation, depression, pregnancy, pregnant, antenatal

## Abstract

Major depression is the most frequent morbidity in pregnancy. The first-line therapies, psychopharmacologic treatment and psychotherapy, are either insufficient or may cause severe or teratogenic adverse events. As a result of its local limitation to the patient’s brain, transcranial direct current stimulation (tDCS) could potentially be an ideal treatment for pregnant women with depression. A literature search was conducted in medical databases, globally published newspapers, search engines, and clinical trial registers to collect all articles on tDCS for the treatment of depression during pregnancy. The aim of this review was to investigate the scientific evidence of tDCS use for depression during pregnancy and to compare these results with the textual and emotional perception in the media as interventions during pregnancy are under particular surveillance. We detected 13 medical articles dealing with tDCS for depression in pregnancy. Overall, the scientific evidence as well as articles in the media for tDCS in pregnancy are sparse, but promising. Further studies are required in this specifically vulnerable population of pregnant women to generate evidence. It is likely that public interest will increase when the results of a pilot study in Canada are published.

## 1. Introduction

The worldwide prevalence of major depression in pregnant women is estimated at up to 10% [1] and is the most frequent morbidity in pregnancy [2]. Untreated antenatal depression may pose risks to the mother and fetus. Small neonates for gestational age, premature delivery, small head circumference, reduced birth weight, and low Apgar scores [3,4] are associated with untreated depression during pregnancy, as well as the higher risk of mental disorders in childhood. For pregnant women, depression during pregnancy is also strongly linked to the development of postpartum depression [5,6]. Lack of treatment or incomplete treatment can cause a higher probability of mental health problems for the unborn child. Treatment options for depression during pregnancy are limited; current guidelines suggest psychopharmacologic treatment and psychotherapy [7,8]. Antidepressant medication (serotonin reuptake inhibitors as a first-line treatment) is effective, but is accompanied by the elevated risk of fetal abnormalities. Neonatal pulmonary hypertension and cardiovascular malformations, reduced birth weight, prematurity, spontaneous abortions, and fetal death are related to medication exposure in pregnancy [2,9,10,11,12]. Although psychopharmacologic treatment is the established first-line treatment of major depression during pregnancy, it is often denied by patients because of fear of fetal abnormalities.

Psychotherapy as monotherapy for episodes of moderate to severe depression is rather ineffective as months of treatment are necessary to improve depression symptoms [8,13], however, counselling can be applied for acute and transient crisis and individual treatment with psychotherapy is feasible in patients with mild depression. Taken together, there is a need for an effective treatment without risks for mother and child.

Reduced neuroplasticity and changed neurocircuitry activity in the prefrontal dorsolateral cortex (DLPFC), that is, hypofunction of the left dorsolateral prefrontal cortex and dysfunctional fronto-limbic control mechanisms, play a significant role in the onset and development of depression [14]. This brain area constitutes an important node of the dysfunctional ‘cognitive control’ network in depression [15]. In recent years, brain stimulation techniques have been shown to exert antidepressant effects by regulating neural plasticity [16]. Repetitive transcranial magnetic stimulation (rTMS) and transcranial direct current stimulation (tDCS) are the most prominent non-invasive brain stimulation methods on the cusp of becoming a third track beside psychopharmacology and psychotherapy in a variety of psychiatric disorders [17,18]. In comparison to other non-invasive brain stimulation methods, for example, rTMS, tDCS is convincing because of its characteristics of low-cost, is simple to handle [19], portable, and especially well tolerated by subjects because of its mild side effects [20]. Furthermore, tDCS has no risk of seizure, which has to be taken into account when applying rTMS [21]. This novel brain stimulation technique has been used for a couple of years as treatment for depression [22].

The rationale behind using tDCS is based on the understanding that modified neuronal activity in the left and right DLPFC including the pathophysiologic models of left hypofrontality and interhemispheric imbalance contributes to the development of depressive disorders [2,23,24]. tDCS is supposed to modulate prefrontal dysfunction by changing local neural activity and activity in remote areas via neural networks [25].

In a simplified model, a constant low current (0.5–2 mA) is transferred through the scalp by two electrodes (anode and cathode) and modulates the neuronal activity depending on the polarity. The brain region under the cathode shows reduced excitability, while the anode enhances excitability [16]. Several studies have reported that tDCS can be judged as a safe method [19] that is compatible with other treatments such as psychopharmacology [26] or psychotherapy [27]. Seminal studies in rats have revealed no injury of brain tissue after tDCS application and more than 33,000 sessions in 1000 humans treated with repeated tDCS sessions showed no serious adverse events or irreversible injury attributed to tDCS [28]. Typical side effects are mild temporary headache during the stimulation sessions, skin sensations under the electrode, or pruritus. These side effects are transient and well tolerated by most patients [29]. Furthermore, tDCS effects are limited to the brain. As there are no systemic effects, tDCS could potentially be an ideal treatment for women with depression during pregnancy. The aim of this review was to investigate the scientific evidence of tDCS use for depression during pregnancy and to compare these results with findings in the newspapers and online sources to investigate the perception in the mass media as medical interventions during pregnancy are under particular surveillance and the application of experimental treatments could be judged as stigmatizing, detrimental, or even taboo.

## 2. Methods

A literature search was conducted without any time frame through medical databases, globally published newspapers, search engines, and clinical trial registers to collect all articles on tDCS for the treatment of depression during pregnancy. Key search words included *1 “transcranial direct current stimulation”, “tDCS”, and “non-invasive brain stimulation” in a cross combination with the term *2 “depression” and *3 “pregnancy” or “antenatal”. Only articles published in English were included. We chose three international, grand standing newspapers with a large online section from three continents (Europe: ‘The London Times’; North America: ‘New York Times’; Asia: ‘China Daily’) to obtain a representative coverage of the topic. The search engines Yahoo and Bing, as well as The London Times newspaper, were excluded from the search as there was no possibility of limiting the hits by advanced search methods. The numerous hits in the Google search engine and the New York Times were limited to the first 20 hits to exclude duplicates and repetition. The search and selection of papers was performed independently by two authors (A.K.K. and U.P.) and, in cases of discrepancy, were controlled by a third author (F.P.). Table 1 shows the hits for the different media.

**Figure 1:**
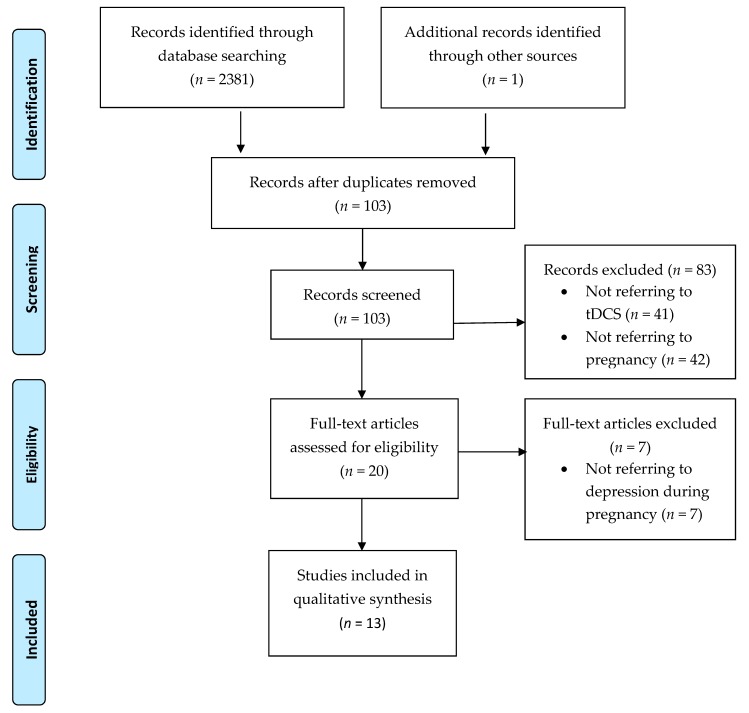
PRISMA flow diagram. tDCS—transcranial direct current stimulation.

An overview over the retrieved literature and its processing is given in the PRISMA (Preferred Reporting Items for Systematic Reviews and Meta-Analyses) flowchart (Figure 1).

## 3. Results

### 3.1. Search Results

Using the keywords and cross combinations previously mentioned, we retrieved 2381 articles. One publication was identified through a manual search. After removing the duplicates, 103 records remained, of which 83 were excluded after being screened. The reasons for exclusion were that most articles referred to the use of brain stimulation techniques other than tDCS for the treatment of depression and were reported on a non-pregnant study population. Articles dealing with tDCS in pregnancy were rare. In addition, postnatal depression was the subject of several articles, that is, those retrieved through search engines. In total, 13 articles dealing with tDCS in pregnancy were included in the qualitative synthesis. We found six papers in scientific journals, no articles in newspapers, six articles in search engines, and two studies in trial registers. Only two scientific publications described the application of tDCS as a treatment for depression in pregnant women [13,30]. One paper described tDCS as a treatment for auditory hallucinations in schizophrenia during pregnancy, also measuring depression symptoms [31]. Five merely mentioned tDCS as a treatment for depression within a pregnancy context. Three articles referred to a pilot study by Vigod et al. [2,32,33] at the Mount Sinai Hospital, Toronto, Canada, which has been completed and whose results are expected to be published during the course of the year.

### 3.2. Current Scientific Evidence

A search in scientific databases revealed six articles. Only three of these publications reported the use of tDCS during pregnancy. In Bangalore, India, a pregnant woman was successfully treated with tDCS. This was the first report of a tDCS application in a pregnant woman with depression. The stimulation was performed with a direct current of 2 mA daily for 10 days, with the anode placed over the left and the cathode placed over the right DLPFC. At the end of the follow-up-phase, the Hamilton Depression Scale (HAMD) changed from 18 to 5 points and the Hamilton Anxiety Rating Scale (HAMA) changed from 32 before intervention to 6 after intervention, both showing remission [13]. Another case report of tDCS as monotherapy for auditory hallucinations in schizophrenia during pregnancy mentions the impact of tDCS on depression symptoms after twice-daily tDCS (20 sessions in 10 days, stimulations within three hours in the morning) with the anode over the left DLPFC and the cathode over the left temporoparietal junction. Based on the Calgary Depression Scale in Schizophrenia (CDSS), depression symptoms associated with schizophrenia were reduced by 41%, although the primary treatment intention was to improve auditory hallucinations [31]. Two review articles were on neuromodulation as a treatment for depression, presenting tDCS as a promising, but still investigational intervention [34,35] The Canadian pilot study is a first step in this direction [2]. Current evidence from studies including reports on side effects is summarized in Table 2.

### 3.3. Trial Register Search

The abstract search on ClinicalTrials.gov and ICTRP (International Clinical Trials Register Platform; World Health Organization, WHO) found one recruiting and one completed study dealing with tDCS for depression during pregnancy. The currently recruiting, single-center, interventional study aims to assess the use of tDCS in pregnant women with major depressive disorder. A target sample size of 10 participants are intended to receive 20 active tDCS sessions (intensity 2 mA) within two weeks, followed by an optional second phase of two weeks with once daily stimulation at the Department of Psychiatry and Psychotherapy, Ludwig Maximilian University in Munich, Germany (DRKS00008537). The preliminary results have been published as a congress proceeding [30], reporting on the promising results of an enhanced tDCS protocol with 30 stimulations within four weeks.

A multi-center, pilot randomized controlled trial, whose recruitment has already been completed aimed to investigate the effect of 15 tDCS sessions within three weeks in 36 pregnant women with major depressive disorder. The patients were treated at the Mount Sinai Hospital Toronto, Canada, for 30 minutes with active 2 mA tDCS [2]. The study results will be published in the near future (NCT02116127).

### 3.4. Results from the Media

Five articles in the media were retrieved from the literature search dealing with tDCS for depression in pregnancy. The fact that no newspaper articles with this main topic could be found may indicate that there is not enough public interest on tDCS for depression during pregnancy yet. However, all articles from the search engines stated with a positive tenor that tDCS could become an adequate and approved treatment option for depression. One article in a tDCS blog described a young woman with depression who was treated as part of the Canadian pilot study at Mount Sinai Hospital, which “brought back life to (her)” [33]. The Perth Brain Centre offered tDCS in the context of two articles. A guide for the treatment of perinatal depression referred to tDCS in several paragraphs and provided a summary on tDCS [36,37]. The Harvard Medical School published an overview on brain stimulation techniques, stating that “tDCS is still considered experimental” [38]. The digital publication Nova Next presented tDCS by citing Simone Vigod, the principal investigator of the pilot study in Canada, who pointed out that tDCS could not only be a useful therapy for pregnant women, but also for treatment resistant depression. This stimulation technique could be even used at home after being approved for efficacy and safety [32].

## 4. Discussion

The object of this review was to identify the scientific evidence on tDCS use for depression during pregnancy and whether there was an echo in the media. The results showed that there is currently sparse scientific literature dealing with tDCS during pregnancy. Two of the six scientific publications were case reports and could merely provide initial suggestions for the future application of tDCS in pregnant women [13,31]. Another ongoing study reported on positive preliminary results [30]. In all of the reviewed articles, no adverse events or severe side effects occurred, that is, no pregnancy-related or fetal complications. However, this is based on only three case reports. These findings are in line with earlier reports on the use of rTMS in depression during pregnancy, where no adverse events were found in several case reports and small studies [39]. Furthermore, follow-up examinations of children being exposed to rTMS during pregnancy revealed no retardation in cognitive or motor development [40]. Electric field modelling could help to predict current distribution during tDCS as already available for rTMS [41].

All screened articles in the search engines mentioned that tDCS, if approved by authorities, could potentially be a suitable therapy option for depression during pregnancy. However, the available evidence is still sparse as large treatment studies with established protocols are lacking or are still under investigation. The forthcoming results of the first randomized controlled trial for tDCS as a treatment for depression during pregnancy with 36 participants [2] will help direct the development of expanded randomized controlled trials, and finally multicenter trials with a focus on efficacy and safety.

This sparse, but promising scientific evidence has also been reflected in the media. Given the few articles, reports for tDCS in pregnancy are lacking. No significant importance has yet been attached to this topic in society except for the Canadian pilot study. This lack of reception is understandable as there is insufficient scientific evidence on treating depression during pregnancy with tDCS up to the present. However, all articles deemed tDCS as a potentially helpful treatment method for depressed women during pregnancy that was “safe and effective” [36] and “potentially life changing” [32]. It is likely that public interest will rise when the results of the randomized clinical study [2] are published.

Generally, it has to be stated that tDCS is a safe and promising treatment option for major depression, as shown in a variety of studies over the past decade [25] including large-scale trials with positive results showing the superiority of tDCS over the placebo [26,42]. Recent meta-analysis has also showed positive results on the safety and acceptability of tDCS in depression [43], and on the cognitive effects [24]. Thus, there is increasing evidence of the benefits of tDCS in distinct symptom domains of depression and that different domains can be used as predictors of response to tDCS [44].

## 5. Conclusions

Depression is the most common morbidity during pregnancy; however, prevalent treatment options are inadequate or insufficient and risk harm to the mother and fetus. tDCS has the potential to develop into a third treatment option for depression during pregnancy. To date, based on available evidence, tDCS is considered as a safe, easy-to-handle, and portable brain stimulation technique in non-pregnant patients. Although tDCS has a favorable safety profile and has been used to treat depression for more than a decade in numerous clinical trials, this review showed that there is currently an insufficient level of evidence for tDCS during pregnancy. Further large-scale studies with longitudinal design are required to assess the safety of tDCS during pregnancy as current safety data refer to non-pregnant patients. In this specifically vulnerable population of pregnant women, non-invasive brain stimulation techniques like tDCS should therefore be explored further, but carefully. This means that, if applying randomized controlled trials, stepped care protocols with active open label phases for those patients allocated to the placebo group could be provided. A thorough assessment of fetal development (e.g., ultrasound) and postnatal surveillance of both the baby and mother in terms of potential side effects related to tDCS should be established as a standard. The pilot study results from Canada will show if larger multicenter trials should be initiated to generate evidence on tDCS as an effective and safe therapy for depression during pregnancy.

## Figures and Tables

**Table 1:** Search strategy and hits.

**Medical databases** PubMed (14 hits) Ovid (9 hits) PsychInfo (8 hits) Embase (29 hits)	**Newspapers/Online Journals** New York Times (1257 hits) China Daily (0 hits) The London Times (>100,000 hits)
**Search engines** Google (1058 hits) Yahoo (>37,700,000 hits) Bing (>35,600,000 hits)	**Trial Register** ICTRP = WHO Trial Platform (4 hits) ClinicalTrials.gov (2 hits)

ICTRP: International Clinical Trials Register Platform; WHO: World Health Organization.

**Table 2:** Characteristics of studies and case reports on transcranial direct current stimulation (tDCS) use in pregnancy.

Author/Journal	Type/Number of Participants/Age/Diagnosis	Electrode Placement/Electrode Size	Stimulation Parameters	Results	Adverse Events/Side Effects/Examinations in Fetus and Mother
Sreeraj et al. Brain Stimulation 2016 [13]	Case report *n* = 1 23 years Recurrent depression	Anode: F3, Cathode: F4 25 cm^2^	2 mA, 30 min/day, 10 days	At the end of the follow-up-phase, HAMD changed from 18 to 5 points and HAMA changed from 32 before intervention to 6 after intervention, both showing a remission.	The patient tolerated tDCS well without any adverse event. In 3 out of the total 10 tDCS sessions, side effects were reported: during the fade-in phase transient, mild burning sensations at the site of application and phosphenes. No specific examinations in fetus/mother.
Strube et al. J Clin Psychopharmacol, 2016 [31]	Case report *n* = 1 36 years Schizophrenia	Anode: F3, Cathode: Tp3	2 mA, 2 × 20 min/day, 10 days	Based on the Calgary Depression Scale in Schizophrenia (CDSS), depression symptoms associated with schizophrenia were reduced by 41% although improvement of auditory hallucinations was the primary target.	tDCS was well tolerated with no reported or noticeable adverse events or side effects. Fetal examination via standard ultrasound at follow-up (gestational week 35) revealed no abnormalities. Normal sonography and delivery 1 week before calculated date.
Palm et al. Clin Neurophysiol, 2017 [30]	Pilot Study (with target sample of *n* = 10) First results: *n* = 3 23, 28 and 32 years (Recurrent) Depression	Anode: F3, Cathode: F4	2 mA, 2 × 30 min/day for 10 days and 1 × 30 min/day for 10 days	No statistically significant changes could be observed yet. One patient achieved remission.	tDCS was well tolerated and no adverse events occurred. No specific examinations in the fetus/mother.

HAMD: Hamilton Depression Rating Scale; HAMA: Hamilton Anxiety Rating Scale; electrode placements according 10–20 EEG system: F3/F4: left/right dorsolateral prefrontal cortex; Tp3: left temporoparietal junction.
